# Safety and safety protocols for living donor nephrectomy in Italy

**DOI:** 10.1007/s13304-023-01678-2

**Published:** 2023-11-08

**Authors:** Niccolò Napoli, Emanuele F. Kauffmann, Michael Ginesini, Cesare Gianfaldoni, Pamela Fiaschetti, Ilaria Lombardi, Massimo Cardillo, Fabio Vistoli, Ugo Boggi

**Affiliations:** 1 Division of General and Transplant Surgery, University of Pisa, Pisa, Italy; 2 Centro Nazionale Trapianti – Italian National Transplant Centre (ISS-CNT), National Institute of Health, Rome, Italy

**Keywords:** Live donor nephrectomy, Live donor renal transplantation, Donor safety, Donor survival, Survey

## Abstract

Living donor kidney transplantation (LDKTx) is recommended by all scientific societies. Living donor nephrectomy (LDN) is probably one of the safest surgical procedures, but it carries some risk for healthy donors. The aim of this study is to provide a snapshot of LDKTx activities in Italy and ask about safety measures implemented in LDN. Data on LDKTx were extracted from the national database. Safety measures were examined through a specific survey. Between 2001 and 2022 40,663 kidney transplants (31.4 per million population-pmp) were performed, including 4731 LDKTx (3.7 pmp). There was no postoperative death of the donor. After a median follow-up of 52.2 months [IQR:17.9–99.5], the 10-year donor survival rate was 93.38% (CI:97.52–98.94). There was evidence of renal disease in 65 donors (1.8%), including 42 (1.1%) with stage III end-stage renal disease. Twenty-nine out of 35 transplant centers (TC) involved in LDKTx responded to the survey (82.9%). Six TCs (21.4%) had a total experience of 20 or fewer LDN. Minimally invasive LDN was the first choice at 24 TC (82.8%). At 10 TC (37.0%) only one surgeon performed LDN. Nineteen TCs (65.5%) had a surgical safety checklist for LDN and 14 had a postoperative surveillance protocol. The renal artery was occluded in 3 TCs (10.3%) mainly by non-transfixion methods (including clips). Redundancy of key safety systems in the operating room was available in 22 of 29 centers (75.8%). In summary, LDKTx should be further implemented in Italy. Donor safety should be improved through the implementation of a national procedural protocol.

## Introduction

Since the first successful procedure between identical twins [1], more than half a million living donor kidney transplants (LKDTx) have been performed worldwide [2]. LKDTx is currently supported by all scientific societies [3] and is recommended in the practice guidelines for kidney transplantation [4, 5]. From the recipient perspective, LKDTx is the ideal opportunity for kidney transplantation [6, 7]. From the donors’ point of view, living donor nephrectomy (LDN) is a safe procedure. The postoperative risk of donor death is 0.03% [8], which is about five times lower than with a cholecystectomy [9]. In the long term, donors are not expected to perform worse than comparable healthy non-donors [8, 10], but should be carefully monitored as there is a small increased risk of end-stage renal disease [11]. The extreme rarity of both early and late complications occurring after LDN makes it impossible to predict individual outcomes.

LDN’s excellent results cannot justify a simplified approach for LDKTx. Rather, they. Should demand standardization and implementation of strict safety protocols. Severe postoperative complications in living donors can have catastrophic consequences [11]. In 2022 the death of a living liver donor triggered a negative media campaign that resulted in a 30 percent decline in living liver transplants in the United States [12]. The advent of minimally invasive LDN (MI-LDN) improved donor satisfaction [13] and increased the number of LDKTx in the United States [14]. However, minimally invasive techniques could not avoid serious postoperative complications and instead raised additional questions about donor safety [15].

In Italy the first successful kidney transplant was a LDKTx performed in Rome on May 3, 1963. The first laparoscopic LDN (April 27, 2000) and the first robotic LDN (November 22, 2008) were both performed in Pisa [16]. According to a recent report by the European Renal Association, 4.8 LDKTx pmp were performed in Italy in 2018 [17]. Considering that these transplants were performed in 35 different transplant centers, it seems that LDKTx is mostly performed on a small scale in Italy [18]. All Italian transplant centers perform a sufficient number of kidney transplants from deceased donors, however LDN is specific to LDKTx and therefore poses particular safety issues.

Against this background, the Italian Society for Organ and Tissue Transplantation (SITO), with the support of the Centro Nazionale Trapianti (CNT) (the government agency that regulates transplantation activities in Italy), has decided to revise the results of LDKTx in Italy and to promote a national survey on LDN. The results of this study were presented at the 45^th^ National Congress of of SITO (October 23–25, 2022; Trieste, Italy).

## Materials and methods

### National data

Data on LDKTx performed in Italy between 2001 and 2022 were extracted from the national database of the CNT. According to the Italian law on transplants from living donors [19], data reporting to the CNT is mandatory.

### Survey

The survey was designed as a cross-sectional study to examine the current practice of LDN in Italy. Forty-one questions were organized into five subject areas: Feasibility and Safety (Questions 1–8), Center Experience and Approach to LDN (Questions 9–12), MI-LDN (Questions 13–21), Operational Details (Questions 22–36) and Postoperative Donor Care (Questions 37–41).

The surveys were recorded with identification data to avoid double answers, but were anonymized before the final analysis.

The survey was conducted according to the Checklist for Reporting of Survey Studies (CROSS) [20].

### Sample characteristics, study preparation and survey administration

The survey was sent to leaders of kidney transplant programs. Only one response per program was allowed. No exclusion criteria were applied.

The questions were agreed upon by SITO and CNT and the survey was developed on Google Forms (Google, Mountain View, CA, USA), a web-based specialized software. After approval of the final version (August 23, 2022), the survey was first sent out by CNT on September 5, 2022. A remainder was sent out every week for the next four consecutive weeks. The final results were collected on October 18, 2022.

### Statistical analysis

Descriptive statistics and categorical variables were summarized as frequencies, percentages, and rates. The survival of living kidney donors was estimated using the Kaplan–Meier method.

## Results

A total of 73,578 organ transplants were performed in Italy between 2001 and 2022, representing an average annual rate of 58.3 transplants pmp. The annual number of transplants increased from 2793 in 2001 (46.5 transplants pmp) to 3887 in 2022 (64.7 transplants pmp).

**Fig. 1:**
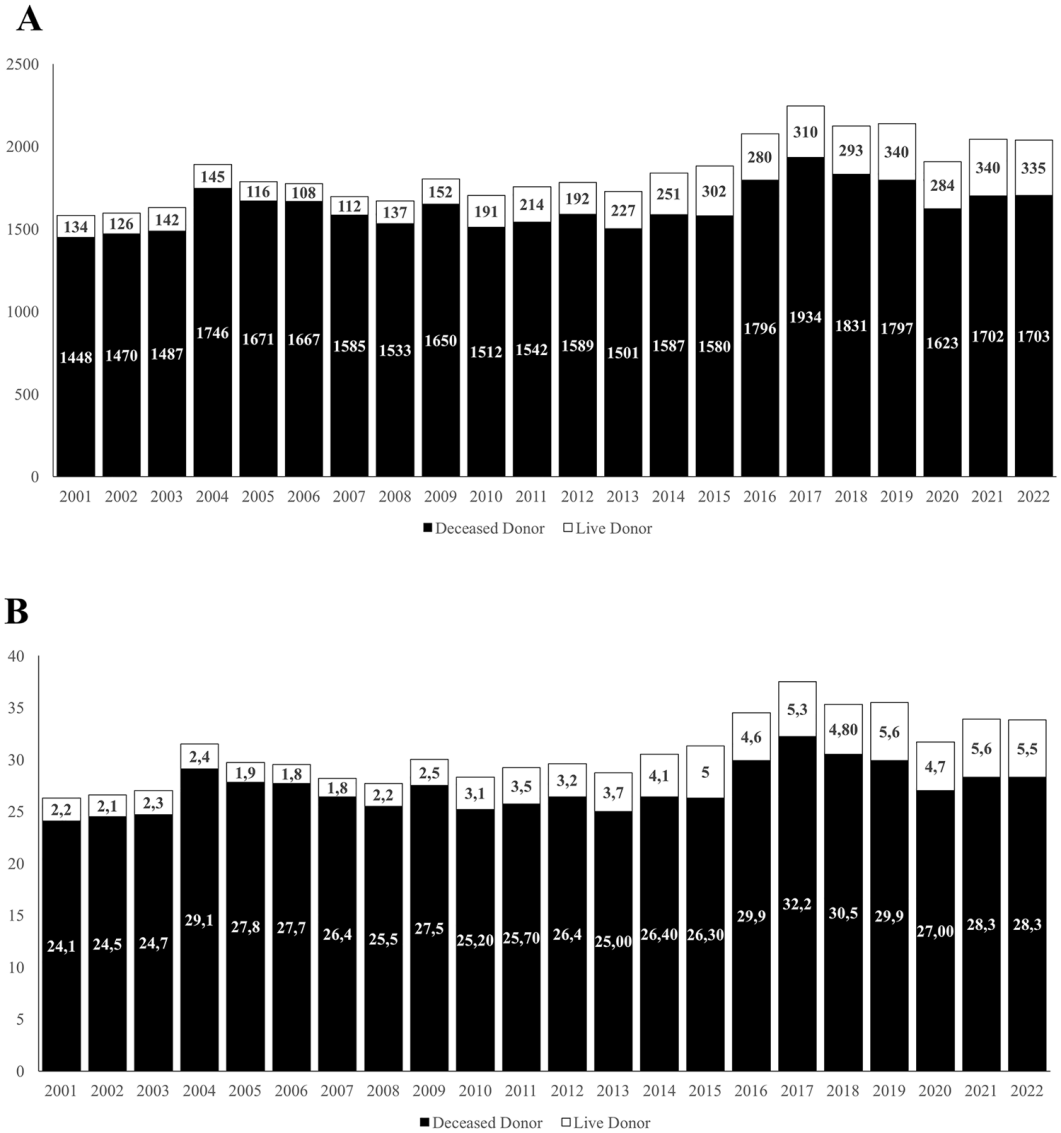
**A.** Annual number of kidney transplants from living (white columns) and deceased (black columns) donors in Italy from 2001 to 2022; **B:** Annual rate per million population of kidney transplants from living (white columns) and deceased (black columns) donors in Italy from 2001 to 2022

Kidney was the most common graft with 40,663 transplants (31.4 pmp), including 35,932 deceased-donor transplants (28.5 pmp) and 4,731 LDKTx (3.7 pmp). The annual number of LDKTx increased from 134 in 2001 (2.4 pmp) to 334 in 2022 (5.6 pmp) (Fig. 1). In 2022, 35 out of 40 transplant centers (92.1%) had an active program for LDKTx. The 40 transplant centers were located in 16 out of 20 Italian regions. LDKTx was carried out in 15 out of 20 Italian regions.

Data on donor characteristics and outcomes were available for LDNs performed between January 1, 2001 and December 31, 2020. During this period, 4010 LDKTx were carried out. The majority of donors were women (68.3%), while the majority of recipients were men (64.6%). Most donors were between 40 and 60 years old (2635; 65.7%). Two donors were under 20 years old (0.04%) and 860 were over 60 years old (2.1%). The mean donor age was 52.5 ± 10.5 years. Blood group O donors were the most common (2303; 57.4%), followed by blood group A (1241; 30.9%), blood group B (397; 9.9%) and blood group AB (69; 1.7%). Most donors were biologically related to their recipients (2547; 63.5%), including mother–child relationships (1119; 43.9%), siblings (607; 23.8%), and father-child relationships. Kinship (501; 20.6%) traded the most typical couples. The remaining 1,436 donors were not biologically related to their recipients. In this group, the typical donor was a spouse (1286; 89.5%).

**Fig. 2:**
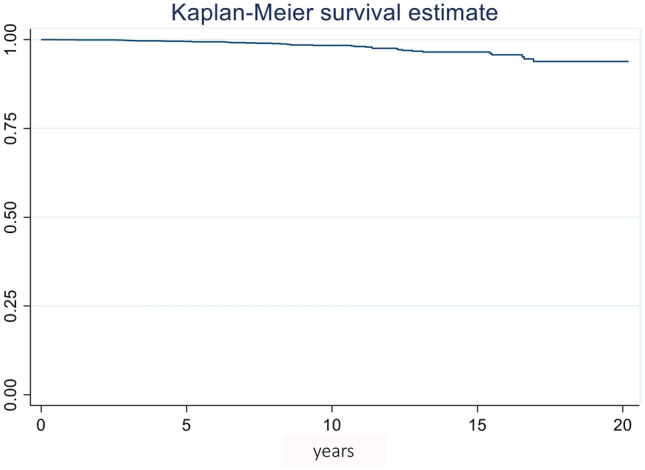
Kaplan–Meier survival curve up to 10 years for living kidney donors

Updated follow-up information was available for 3,551 donors (88.5%). After a minimum follow-up of 8 months (median 65.05 IQR: 33.9–118.6), 3512 donors (98.9%) were alive and 39 (1.0%) had died. The donor survival rate at 10 years was 93.38% (CI: 97.52–98.94) (Fig. 2). The main reasons for donor death were tumors (17; 43.5%) and cardiovascular disease (12; 30.7%). No postoperative death was reported. Fifteen donors died > 10 years after LDN (38.4%), 13 between 5 and 10 years (33.3%) and 9 between 2 and 5 years (27.2%). Two donors died in the first two years after LDN (5.1%).

Sixty-five donors (1.8%) developed some degree of renal disease, including 42 (1.1%) who progressed to end-stage renal disease after a median of 65.3 months (IQR: 24.6–98.3). Five donors (0.14%) required dialysis.

### Survey

**Fig. 3:**
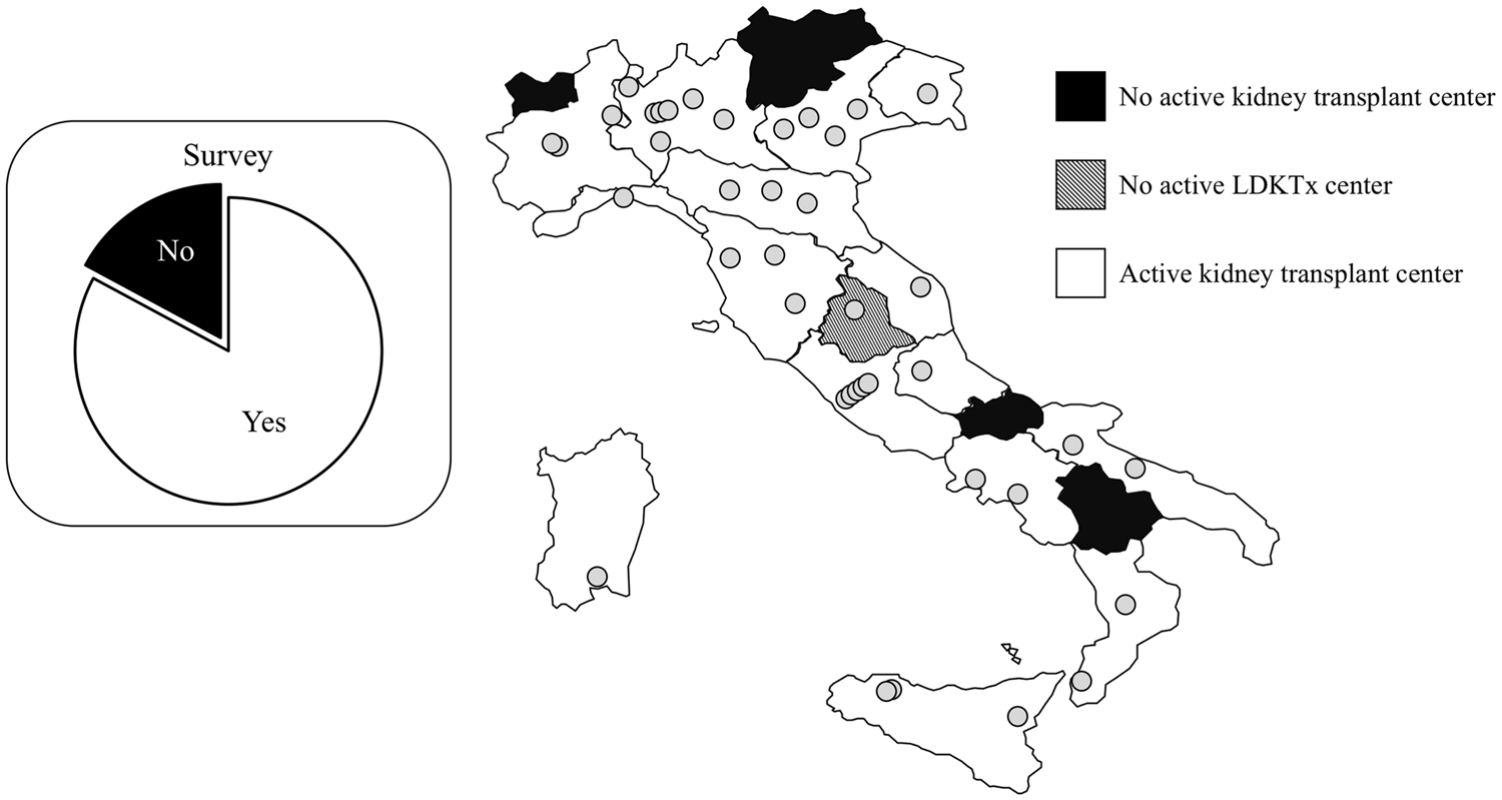
Distribution of 40 kidney transplant centers (circles) in 20 Italian regions. Black regions have no active transplant centers. In white regions with at least one transplant center with an active living donor kidney transplant program. In Umbria (striped region) there is one kidney transplant center that is not active in the transplantation of living donor kidneys. See the square for a graphical representation of the response rate to the survey

Twenty-nine out of 35 transplant centers with an active program for LDKTx responded to the survey (82.9%). Responses came from 13 out of 15 Italian regions with at least one active centre: 11 out of 15 centers (73.3%) from northern Italy, 12 out of 12 centers (100%) from central Italy and 3 out of 4 (75.0%) in both southern Italy and Sicily and Sardinia (Fig. 3). The survey results are summarized in Table 1.

**Table 1:** Summary of survey results

Response	29/35 (82.5%)
Feasibility and safety	
Severe complications resulting in transient disability	1/29 (3.4%)
Severe complications resulting in permanent disability	0
Operative mortality	0
Center experience and approach to LDN	
Time of the first LDN	
before 1990	9/27 (33.3%)
between 1991 and 2009	13/27 (48.1%)
after 2009	5/27 (18.5%)
Overall experience with LDN	
≤ 20	6/29 (21.4%)
21–50	6/29 (21.4%)
51–100	2/29 (7.1%)
101–200	5/29 (17.8%)
201–300	4/29 (14.2%)
301–500	3/29 (10.7%)
> 500	3/29 (10.7%)
Surgeon performing LDN	
General surgeon	27/29 (93.1%)
Urologist	2/29 (6.9%)
Preferred LDN approach	
Minimally invasive	24/29 (82.8%)
Mini-open incision	5/29 (17.2%)
MI-LDN	
First MI-LDN	
In 2000	6/27 (22.2%)
2001–2010	5/27 (18.5%)
2011–2020	11/27 (40.7%)
Missing data	5/27 (18.5%)
Preferred MI-LDN technique	
Laparoscopy	11/24(37.9%)
Robotic assistance	7/24 (24.1%)
Hand-assisted MI-LDN	6/24 (20.7%)
Centers that had performed at least 1 MI-LDN	27/29 (93.1%)
Centers that had performed at least 1 laparoscopic LDN	16/29 (55.2%)
Centers that had performed at least 1 hand-assisted laparoscopic LDN	14/29 (43.3%)
Centers that had performed at least 1 robotic-assisted LDN	18/29 (66.7%)
Centers routinely or selectively using a flank incision for contemporary LDN	12/29 (41.4%)
Availability of a robotic platform	27/29 (93.1%)
First robotic LDN after the year 2020	5/27 (18.5%)
Surgical teams performing LDN through the years	
1	23/27 (79.3%)
> 1	4/27 (13.8%)
Surgeon performing MI-LDN	
Member of the transplant team	22/27 (81.5%)
MI surgeon not involved in transplantation	2/27 (7.4%)
Missing information	3/27 (11.1%)
Surgeons able to perform MI-LDN per center	
1	10/27 (37.0%)
2	13/27 (48.1%)
3	3/27 (11.1%)
> 3	1/27 (3.7%)
Surgeons with the larger experience in MI-LDN per center	
< 20	7/27 (25.9%)
21–50	7/27 (25.9%)
51–100	4/27 (14.8%)
101–200	7/27 (25.9%)
> 200	2/27 (7.4%)
MI experience of surgeons performing MI-LDN per center	
MI nephrectomy	26/27 (96.2%)
MI cholecystectomy	23/27 (79.3%)
MI adrenalectomy	19/27 (70.3%)
MI colectomy	15/27 (55.5%)
MI renal transplantation	8/27 (29.6%)
MI total gastrectomy	7/27 (25.9%)
MI pancreatoduodenectomy	5/27 (18.5%)
Training programs for LDN	
Observation followed by tutored implementation	17/27 (62.9%)
Observation, simulation, and tutored implementation	4/27 (14.8%)
No specific training program	6/27 (22.2%)
Operative details	
Safety check list	
Safety check list specific to LDN	19/29 (65.5%)
Standard safety check list	10/29 (34.4%)
Donor monitoring	
Electrocardiogram and urinary output	28/28 (100%)
Arterial line	18/28 (64.2%)
Central venous line	14/28 (50.0%)
*Redundancy* of standard safety systems	22/29 (75.8%)
Immediate availability of surgical instruments to control major bleeding	
Laparoscopic/robotic vascular clamps	26/27(96.3%)
Instruments for emergency conversion to open surgey	26/27(96.3%)
Source of CO_2_ supply	
Continuous, centralized, supply	5/27 (18.5%)
Portable tanks	8/27 (29.6%)
Combination thereof	14/27 (51.8%)
Immediate availability of bood transfusions in case of emergency need	14/29 (75.8%)
Cell saver	
Routine use	2/29 (6.9%)
Immediately available, if needed	22/29 (75.8%)
Not immediately available	5/29 (17.2%)
Stapler malfunction	9/28 (32.1%)
Consequences of stapler malfunction	
Conversion to open surgery	2/9 (22.2%)
Conversion from laparoscopy to hand-assistance	1/9 (11.1%)
Bleeding	4/9 (44.4%)
Required blood transfusions	1/9 (11.1%)
Closure of renal artery	
Stapling alone	22/29 (75.8%)
Stapling plus polymer ligating clips	3/29 (10.3%)
Stapling plus standard vascular clips	1/29 (3.4%)
Polymer ligating clips	1/29 (3.4%)
Standard vascular clips	1/29 (3.4%)
Standard Ligature	1/29 (3.4%)
Closure of renal vein	
Stapling alone	25 (86.2%)
Stapling plus polymer ligating clips	2 (6.9%)
Polymer ligating clips	1/29 (3.4%)
Standard ligature	1/29 (3.4%)
Postoperative donor care	
Protocol for postoperative surveillance	
Surveillance protocol specific to LDN	14/29 (48.3%)
Standard surveillance protocol	15/29 (51.3%)
Protocol for postoperative analgesia	
Analgesia protocol specific to LDN	12/29 (41.3%)
Standard analgesia protocol	17/29 (58.6%)
Intensity of immediate postoperative care	
General ward	17/29 (58.6%)
High-dependency unit	9/29 (31.0%)
Intensive care unit	3/29 (10.3%)
Timing of hospital discharge (uneventful postoperative course)	
Postoperative day 2	2/29 (6.9%)
Postoperative day 3	10/29 (34.5%)
Postoperative day 4	9/29 31.0%)
Postoperative day 5	7/29 (24.1%)
Postoperative day ≥ 6	1/29 (3.4%)
Blood tests before hospital discharge	
Blood count	29/29 (100%)
Serum creatinine	29/29 (100%)
Serum electrolytes	27/29 (93.1%)
Abdominal ultrasound before hospital discharge	17/29 (58.6%)

One center reported a case in which severe postoperative complications resulted in temporary disability. No case of permanent disability or postoperative death was reported. However, one center (3.4%) reported one death within the first six months after donation. The donor's cause of death was not given, but the death was not attributed to LDN.

The overall experience with LDN was ≤ 100 procedures in approximately 50% of the Italian transplant centers (14/29; 48.2%). Twenty-seven of 29 centers (82.8%) had experience with MI-LDN, but a conventional approach (adapted to a mini-incision) was preferred in 5 centers (17.2%). In 12 centers (41.4%) open LDN was still the preferred donor procedure. Over 50% of centers performed their first MI-LDN after 2010 (16/17; 59.2%). Regarding the technique used for MI-LDN, laparoscopy (37.9%) was used slightly more often than robotic (24.1%) or hand-assistance (20.7%). In the vast majority of centers, only one (37.0%) or two surgeons (48.1%) were skilled in performing MI-LDN, and experience with MI-LDN was limited to 50 procedures in 14 out of 27 transplant centers (51.8%).

Operative procedures showed a quite ample variability. It may be worth to note that two centers (6.9%) used only clips to secure the renal artery, either standard clips or polymer ligating clips, and that a center declared lack of immediate availability of life-saving instruments in the operating room (such as minimally invasive vascular clamps and instruments for immediate conversion to open-LDN) in case of emergency need.

Operative procedures showed a fairly large variability. It may be worth noting that two centers (6.9%) exclusively used clips to secure the renal artery, either standard clips or polymer ligating clips, and one center reported that life-saving tools were not readily available in the operating room (e.g. minimally invasive vascular clamps and instruments for immediate conversion to open-LDN) in an emergency.

With regard to postoperative care, more than 50% of the centers did not have a specific protocol. After the operation, the majority of patients (58.6%) were admitted to a general ward.

## Discussion

The ever-widening gap between demand and supply of kidney grafts makes LDKTx a key element for all transplant centers. However, LDN puts healthy people at risk of surgery and the possible consequences of reduced nephron mass. The risk of donor death is approximately 0.03% (i.e. three deaths per 10,000 LDN) [8], while LDN increases the likelihood of end-stage renal disease by < 0.5% at 15 years [11]. The combination of donor shortage, excellent recipient outcomes and low donor risks provides a solid ethical justification for LDKTx [12]. However, the consequences of severe donor complications should not be underestimated [11, 12]. Standard safety measures may not be sufficient to prevent extremely rare events such as the death of a living kidney donor. On the contrary, the normally smooth flow of LDN could create a false sense of security and reduce individual and team perceptions of potential danger.

This study was specifically designed to provide a snapshot of LDKTx activities in Italy and examine the implementation of safety measures in LDN. The overall picture shows excellent donor results. However, it also shows that LDKTx is performed at low volume without standardizing donor safety.

There is clearly a need to increase the number of LDKTx in Italy. A recent study shows that in 2018 several European countries had higher LDKTx rates pmp than Italy (4.8). These countries include: Portugal (5.7), Finland (5.8), Spain (6.3), Greece (6.6), Macedonia (7.1), Germany (7.7), Austria (7.9), Montenegro (8.0), France (8.1), Ireland (8.2), Malta (8.3), Albania (9.1), Latvia (9.9), Denmark (13.2), Switzerland (13.3), Norway (13.7), Sweden (14.0), United Kingdom (15.3), Cyprus (17.2), Iceland (25.5), The Netherlands (28.1), Turkey (37.0). In some of these countries, LKDTx donation rates may not be comparable to those in Italy due to smaller populations and/or different socioeconomic and cultural conditions. However, some countries are actually comparable to Italy (e.g. Spain, Germany, France, Switzerland, the United Kingdom and the Netherlands). These countries not only had higher rates of LKDTx, but also higher rates of kidney transplantation from deceased donors (Italy: 30.3 pmp; France: 45.2 pmp; Spain: 64.6 pmp) [17]. These figures clearly show the need for a standardized approach to LDKTx in Italy with the aim of increasing this activity. The Dutch or Israeli model could be an option [21, 22] or a new model could be designed and implemented. To make such a national program successful, it is necessary to ensure solid political and economic support. The experience with the Tuscan model of organ donation from deceased donors shows the importance of political and economic support [23].

An increase in LDKTx activity could further increase the safety of LDN. In fact, the survey found that about half of the transplant centers had an overall experience of less than 100 LDN and 20% had an overall experience of less than 20 LDN. These small volumes have obvious implications for training, as evidenced by the fact that in over a third of transplant centers only one surgeon was able to perform a LDN. The fact that neither the national data nor the survey revealed any actual donor safety concerns may reflect the rarity of severe complications after LDN and should not be taken as conclusive evidence of absolute safety.

Volume-safety interactions have been demonstrated for many surgical procedures, although the figures for complex surgeries were more striking [24]. In general, the volume-outcome paradigm applies to all surgical procedures and should particularly apply to LDN due to the devastating consequences of each single serious adverse event. For this reason, the American Society of Transplantation Live Donor Community of Practice developed a crisis management plan for living donors [25]. The Organ Procurement Network recommends 15 LDN before indepedent practice [26]. This number of interventions could not be easily performed in about one in five Italian transplant centers. In addition, a study on training in LDN showed that 35 procedures were required to overcome the learning curve, based on operative time. Improved clinical outcomes (for both donors and recipients) were obtained with a median annual volume of 50 LDN [27]. Whether not meeting these volume standards should pose a barrier to individual surgeons and transplant centers performing LDN is not the aim of this discussion. However, it is clear that the number of LDKTx should be increased. Higher volume of activity is expected to increase the safety of LDN and possibly the outcome of LDKTx.

With regard to donor safety, the survey provided further important information. First, despite clear evidence that clips alone cannot be used to occlude renal vessels in LDN, few centers considered clips their first choice to control renal arteries. Second, most LDNs are performed using standard safety measures. Third, postoperative surveillance in LDN is not improved compared to standard surgery.

The landmark study by Friedman and co-workers clearly showed that arterial bleeding poses a risk to donor safety, particularly when it occurs in the postoperative period. Clips were used in 25 of 66 donors (37.8%) who experienced life-threatening arterial bleeding. The failure rate was similar regardless of the clip used (ligating clips: 12/66; 18.1%) (conventional hemostatic clips: 13/66; 19.6%). More generally, this study showed that 45 out of 66 donors (68.1%) who developed massive arterial bleeding after LDN had the renal arteries occluded by a method without transfixion (e.g., clips) [15]. Based on these data, the manufacturer (Teleflex, Wayne, PA, USA) stated in the package insert that ligating clips *“are contraindicated for renal artery ligation during laparoscopic donor nephrectomy”* [28]. The fact that the few Italian centers continue to use either standard or ligating clips for primary renal artery control raises serious concerns about donor safety.

If delayed, massive arterial bleeding is more likely to be fatal or serious, underscoring the importance of postoperative monitoring. In the study by Friedman et al., inadequate pain control was the cause of delayed arterial bleeding in at least one patient [15]. The survey revealed a lack of standardization in protocols for postoperative surveillance of living kidney donors.

The survey also revealed a lack of redundancy in key technology in the operating room, which could lead to serious clinical consequences in the event of equipment failure. Although the principle of safety redundancy should be applied to all surgical procedures, it appears to be particularly important in LDN. Technology redundancy should be standard at LDN.

This study has several limitations. First, the data provided by CNT were prospectively entered into a national database but analyzed retrospectively, introducing some of the biases typically associated with this study design. Second, the data required by CNT and stored in the Sistema Informativo Trapianti may not be sufficient to capture all early and late adverse events that may occur after LDN. In particular, follow-up should be lifelong and carefully cover all medical aspects that may be related to kidney donation. Therefore, some complications and long-term consequences of LDN may have been underestimated in this study. Based on an expected fatality rate of 0.03%, the safety profile of LDN could only be verified in a prospective observational study involving over 15,000 procedures.

Such a study in Italy would take several decades and would likely be biased by time-dependent variables. Third, the survey tool itself has inherent limitations. Fourth, despite the high response rate (82.9%), some centers did not respond. The reasons for missed responses are not clear but could be the lack of standardized safety protocols and/or unsatisfactory results. Fifth, some centers did not respond to some questions, possibly reflecting concerns about the center’s policies on specific questions raised in the survey.

In summary, LDKTx should be further implemented in Italy. To this end, a national plan is urgently needed. Donor safety could be further improved by increasing annual activity and introducing a national safety protocol. Privileges for LDN should only be granted after passing a specific test and should only be maintained if competence is demonstrated. Privileges for LDN should not only be granted to surgeons, but rather to the entire surgical team, including anesthesiologists and surgical nurses.

## Data Availability

All materials are available upon request.
